# Association of serum 25-hydroxyvitamin D concentrations with risk of dementia among individuals with type 2 diabetes: A cohort study in the UK Biobank

**DOI:** 10.1371/journal.pmed.1003906

**Published:** 2022-01-13

**Authors:** Tingting Geng, Qi Lu, Zhenzhen Wan, Jingyu Guo, Liegang Liu, An Pan, Gang Liu

**Affiliations:** 1 Department of Nutrition and Food Hygiene, Hubei Key Laboratory of Food Nutrition and Safety, Ministry of Education Key Lab of Environment and Health, and State Key Laboratory of Environmental Health (Incubating), School of Public Health, Tongji Medical College, Huazhong University of Science and Technology, Wuhan, China; 2 Department of Epidemiology and Biostatistics, Ministry of Education Key Laboratory of Environment and Health, School of Public Health, Tongji Medical College, Huazhong University of Science and Technology, Wuhan, China; University of New South Wales, AUSTRALIA

## Abstract

**Background:**

Several epidemiological studies have suggested that vitamin D status is associated with risk of dementia in general populations. However, due to the synergistic effect between diabetic pathology and neuroinflammation, and the prothrombotic profile in patients with diabetes, whether vitamin D is associated with risk of dementia among patients with diabetes is unclear. This study aimed to investigate the associations of circulating vitamin D levels with risks of all-cause dementia, Alzheimer disease (AD), and vascular dementia (VD) among adults with type 2 diabetes (T2D).

**Methods and findings:**

This study included 13,486 individuals (≥60 years) with T2D and free of dementia at recruitment (2006–2010) from the UK Biobank study. Serum 25-hydroxyvitamin D (25[OH]D) concentrations were measured using the chemiluminescent immunoassay method at recruitment. Serum 25(OH)D ≥ 75 nmol/L was considered sufficient, according to the Endocrine Society Clinical Practice Guidelines. Incidence of all-cause dementia, AD, and VD cases was ascertained using electronic health records (EHRs). Each participant’s person-years at risk were calculated from the date of recruitment to the date that dementia was reported, date of death, date of loss to follow-up, or 28 February 2018, whichever occurred first. Among the 13,486 individuals with T2D (mean age, 64.6 years; men, 64.3%), 38.3% had vitamin D ≥ 50 nmol/L and only 9.1% had vitamin D ≥ 75 nmol/L. During a mean follow-up of 8.5 years, we observed 283 cases of all-cause dementia, including 101 AD and 97 VD cases. Restricted cubic spline analysis demonstrated a nonlinear relationship between serum 25(OH)D and risk of all-cause dementia (*P*_nonlinearity_ < 0.001) and VD (*P*_nonlinearity_ = 0.007), and the nonlinear association reached borderline significance for AD (*P*_nonlinearity_ = 0.06), with a threshold at around a serum 25(OH)D value of 50 nmol/L for all the outcomes. Higher serum levels of 25(OH)D were significantly associated with a lower risk of all-cause dementia, AD, and VD. The multivariate hazard ratios and 95% confidence intervals for participants who had serum 25(OH)D ≥ 50 nmol/L, compared with those who were severely deficient (25[OH]D < 25 nmol/L), were 0.41 (0.29–0.60) for all-cause dementia (*P*_trend_ < 0.001), 0.50 (0.27–0.92) for AD (*P*_trend_ = 0.06), and 0.41 (0.22–0.77) for VD (*P*_trend_ = 0.01). The main limitation of the current analysis was the potential underreporting of dementia cases, as the cases were identified via EHRs.

**Conclusions:**

In this study, we observed that higher concentrations of serum 25(OH)D were significantly associated with a lower risk of all-cause dementia, AD, and VD among individuals with T2D. Our findings, if confirmed by replication, may have relevance for dementia prevention strategies that target improving or maintaining serum vitamin D concentrations among patients with T2D.

## Abstract 2

In an observational study, Tingting Geng and colleagues investigate associations between serum vitamin D and dementia risk among individuals with Type 2 diabetes in the UK Biobank.

## Author summary

**Why was this study done?:**

- Compared with the general populations, individuals with diabetes are particularly susceptible to both vitamin D deficiency and increased risk of developing dementia.
- Although some epidemiological studies have linked lower vitamin D concentrations with risk of dementia in general populations, the evidence regarding the relationship between vitamin D status and dementia risk among patients with diabetes is scarce.

**What did the researchers do and find?:**

- We conducted an analysis using data from the UK Biobank study to examine the association of serum vitamin D levels with risk of dementia among 13,486 patients with type 2 diabetes who were ≥60 years old.
- Higher serum levels of vitamin D were significantly associated with a lower risk of dementia.

**What do these findings mean?:**

- We observed that vitamin D status may be associated with risk of all-cause dementia, vascular dementia, and Alzheimer disease, and this is important as our data suggest that vitamin D deficiency (serum 25[OH]D < 50 nmol/L) is common in individuals with diabetes.
- The findings suggest the possibility that serum vitamin D screening among patients with diabetes may be useful in dementia care and prevention.

## Introduction

Type 2 diabetes (T2D) is a serious public health issue associated with high morbidity and mortality rates, imposing a considerable health and economic burden [1]. Prevention of diabetes and related complications is paramount. Increasing evidence has indicated a decline in rates of vascular disease mortality, which leads to a diversification of diabetes-related mortality [2,3]. UK Office for National Statistics mortality data show that 10 causes of death among diabetic patients have declined; however, death rates due to dementia have increased [2]. Therefore, the strategies for dementia prevention should be particularly emphasized among individuals with diabetes.

Several epidemiological studies have linked serum vitamin D with risk of dementia in general populations [4–7]. Although the mechanism has not been fully elucidated, it is widely postulated that vitamin D may reduce dementia risk by mitigating neuroinflammation and vascular damage [8–10]. However, due to the potential synergistic effect between diabetic pathology and neuroinflammation [11], and the prothrombotic profile in patients with diabetes [12], whether the potential favorable effect of circulating vitamin D on dementia risk can be extrapolated to patients with T2D has yet to be elucidated. Moreover, compared with general populations, individuals with diabetes are particularly susceptible to both vitamin D deficiency [13,14] and increased risk of developing dementia [15,16]. Although several cross-sectional and case–control studies have suggested an inverse association between serum 25-hydroxyvitamin D (25[OH]D) and cognitive function decline assessed using neuropsychological tests among patients with T2D [17–19], these studies had limitations of small sample size, low quality of study design, and insufficient adjustment for critical confounders such as *apolipoprotein E (APOE) ε4* genotype, severity of diabetes, and lifestyle factors.

To fill these knowledge gaps, we examined the association of serum 25(OH)D concentration with the risk of all-cause dementia, Alzheimer disease (AD), and vascular dementia (VD) among 13,486 individuals with T2D who were ≥60 years old in the UK Biobank study.

## Methods

### Study population

The UK Biobank is a large population-based prospective cohort study for long-term study of genetic and lifestyle determinants of a wide range of common diseases of middle and old age. The design of the UK Biobank has been presented previously [20]. Briefly, it recruited more than 500,000 participants (aged 40–69 years) from 22 assessment centers across England, Scotland, and Wales between 2006 and 2010. Extensive phenotypic and genotypic data were collected at recruitment. Information on socio-demographics, habitual diet, lifestyle factors, and medical history was collected through touch-screen questionnaires; anthropometric data were obtained through physical measurements. Blood, urine, and saliva samples were also collected at baseline in all participants.

In the present study, we included 23,748 prevalent T2D cases at recruitment. Prevalent cases of T2D were identified using a UK Biobank algorithm that identified cases through multiple sources including self-report, trained health professional queried medical history, and medication history, which has been shown to be a reliable measurement, with 96% accuracy [21]. Overall, we included data from 13,486 participants who were ≥60 years old in the main analysis after excluding participants who had a previous diagnosis of dementia (*N* = 18), patients with incomplete information on serum 25(OH)D concentration (*N* = 2,096), and cases that were identified as involving death from dementia in the death registry (*N* = 12), to minimize the effect of VD on survival (S1 Fig).

The UK Biobank study was approved by the National Information Governance Board for Health and Social Care in England and Wales, the Community Health Index Advisory Group in Scotland, and the North West Multi-centre Research Ethics Committee. All participants gave written informed consent. This study is reported as per the Strengthening the Reporting of Observational Studies in Epidemiology (STROBE) guideline (S1 Checklist). Our prospective analysis plan is included in (S1 Text).

### Assessment of serum 25(OH)D

Blood samples were collected from consenting participants at recruitment, separated by components, and stored at UK Biobank (−80°C and liquid nitrogen) until analysis. Serum concentration of 25(OH)D (nmol/L) was measured using the chemiluminescent immunoassay method (DiaSorin Liaison XL). The assay’s analytical range was between 10 nmol/L and 375 nmol/L. The coefficient of variation for serum 25(OH)D ranged between 5.04% and 6.14%, and the results of the external quality assurance were 100%. Full details on assay performance have been published previously [22].

### Ascertainment of incident dementia cases

Dementia cases were ascertained using the algorithms provided by UK Biobank, which were generated based on electronic health records (EHRs) including hospital admissions and the death registry, using ICD-9 and ICD-10 codes (S1 Table). Medical history information on dementia-specific prescriptions including memantine, donepezil, galantamine, and rivastigmine was also used to identify prevalent dementia cases at baseline [23].

### Assessment of covariates

Information on height and body weight was collected during a nurse-led interview. Body mass index (BMI, kg/m^2^) was calculated as body weight in kilograms divided by the square of height in meters. Physical activity was assessed using the short-form International Physical Activity Questionnaire (IPAQ) including walking and moderate and vigorous intensity activities. Total physical activity in metabolic equivalent minutes per week (MET-min/week) was computed from the IPAQ. Information on habitual diet and alcohol intake was captured by a touchscreen food frequency questionnaire at recruitment in all participants. A hypothesis-driven dietary pattern was computed based on 5 components to reflect the overall diet. Participants received points for consumption in 4 healthy food categories (fruits, vegetables, whole grains, and low-fat dairy) based on quintile (from 5 points in the highest quintile to 1 point in the lowest quintile). The category for unhealthy food (red and processed meat) was inversely scored. The overall diet score then was categorized into quintiles. Data on medication history were collected via interview. If the participants were not sure about the types of medications they were taking, they were asked to provide the medications they were taking later in the visit.

### Statistical analysis

Each participant’s person-years were calculated from the date of recruitment to the date of reported dementia diagnosis, death, or loss to follow-up, or 28 February 2018, whichever occurred first. As 16 individuals were diagnosed with both AD and VD, we calculated the person-years for AD and VD separately. We imputed missing values (<5.3%) using multiple imputation by chained equations with 20 imputations. Given that few participants had serum 25(OH)D ≥ 75 nmol/L in the current study population, serum vitamin D status was categorized into 3 groups: severely deficient (<25 nmol/L), moderately deficient (25 to <50 nmol/L), and insufficient and above (≥50 nmol/L), according to the Endocrine Society Clinical Practice Guidelines [24]. Distributions of the baseline characteristics were compared across the categories of the serum 25(OH)D concentration.

We used multivariable Cox proportional hazards regression models to compute hazard ratios (HRs) and 95% confidence intervals (CIs) for the association between serum 25(OH)D concentration and risk of all-cause dementia, AD, and VD. The relationships between serum 25(OH)D level and risk of the outcomes were first evaluated on a continuous scale using restricted cubic spline analysis. We selected the number of knots based on the values of the Akaike information criterion to fit the best-approximating model, chose the lowest value of serum 25(OH)D as reference, and tested for linearity by Wald test.

In Model 1, we adjusted for the following potential confounders: age at recruitment (continuous, years), sex (male, female), education (college or university degree, A/AS levels or equivalent or O levels/General Certificate of Secondary Education [GCSE] or Certificate of Secondary Education or equivalent, National Vocational Qualification or Higher National Diploma or Higher National Certificate or equivalent or other professional qualifications, none of the above), socio-economic status (Townsend deprivation index, continuous), ethnicity (White, Asian, Black, Mixed), blood collection season (Dec–Feb, Mar–May, Jun–Aug, Sep–Nov), sun-exposure time in summer (continuous, hours/day), and *APOE ε4* genotype (carrier, non-carrier). In Model 2, we further adjusted for BMI (continuous, kg/m^2^), alcohol intake (never or special occasions, monthly to weekly, daily), smoking status (never, past, current), physical activity (continuous, MET-hours/week), healthy diet score (in quintiles), sleep duration (≤6, 7–8, ≥9 hours/day), and multivitamin intake (yes, no). In Model 3, we further adjusted for diabetes duration (continuous, years); concentrations of hemoglobin A1c (HbA1c; continuous, mmol/mol), total cholesterol (TC; continuous, mmol/L), triglycerides (TGs; continuous, mmol/L), low-density lipoprotein cholesterol (LDL-C; continuous, mmol/L), and C-reactive protein (CRP; continuous, mg/L); medication for diabetes (none, only oral medicine, insulin and others), medication for hypertension and cholesterol (yes, no); and history of hypertension, cardiovascular disease, cancer, and depression (yes, no). The median values were assigned to each category of serum 25(OH)D concentration to test for linear trends.

We also stratified the analyses by sex (male, female), *APOE ε4* genotype (carrier, non-carrier), BMI (≤30, >30 kg/m^2^), diabetes duration (≤7, >7 years), and smoking status (never, ever). The multiplicative interactions between serum vitamin D status and the stratification factors on the risk of dementia were tested using the likelihood ratio test by including an interaction term.

Several sensitivity analyses were conducted. First, to minimize the possibility of reverse causality in the observed associations, we repeated the analyses excluding participants with less than 2 years of follow-up. Second, as kidney function may influence circulating vitamin D levels and cognitive function, estimated glomerular filtration rate (eGFR) was further adjusted for. Third, as frailty is a potential risk factor of dementia, we further adjusted for walking pace and hearing impairment. Fourth, serum calcium was adjusted for, and finally, vitamin D supplements were additionally adjusted for. Fifth, we also repeated the analysis categorizing serum 25(OH)D values as severely deficient (<25 nmol/L), moderately deficient (25 to <50 nmol/L), insufficient (50 to <75 nmol/L), and sufficient (≥75 nmol/L). In addition, we performed the main analyses in the full cohort including individuals with or without T2D (≥60 years old; *N* = 192,862). Finally, to further clarify the potential effect modification of T2D, we also tested the association of vitamin D with risk of outcomes stratified by T2D status. The last 4 sensitivity analyses were requested by the reviewers, and were not included in the analysis plan.

All analyses were performed using SAS version 9.4 (SAS Institute) and Stata statistical software, release 14.0 (StataCorp, College Station, Texas), and a 2-sided *P* < 0.05 was set as the threshold for statistical significance.

## Results

Among the 13,486 adults with T2D (*N* = 8,668 [64.3%] male), the mean age was 64.6 years, and 2,288 (17.0%) were vitamin D severely deficient (<25 nmol/L), 6,036 (44.8%) were moderately deficient (25 to <50 nmol/L), and 5,162 (38.3%) were insufficient or above (≥50 nmol/L). Of note, only 1,228 (9.1%) participants were vitamin D sufficient (≥75 nmol/L). During a mean follow-up of 8.5 years, we observed 283 incident all-cause dementia cases, including 101 cases of AD and 97 cases of VD. Distributions of baseline characteristics according to the categories of serum 25(OH)D levels are shown in Table 1. Participants with higher concentration of serum 25(OH)D were more likely to be men, less deprived, less educated, White (British, Irish, and any other White background), physically active, daily drinkers, and not current smokers, and were more likely to have a shorter duration of T2D and a lower BMI. They were also more likely to sleep 7-8 hours/day, have a healthier diet, have a lower prevalence of pre-existing hypertension and cardiovascular disease, and have lower levels of circulating TC, TGs, LDL-C, CRP, and HbA1c.

**Table 1:** Baseline characteristics according to serum 25(OH)D concentrations among individuals with type 2 diabetes who were ≥60 years old in the UK Biobank study.

Characteristic	Serum 25(OH)D concentration (nmol/L)			*P* value
	<25	25 to <50	≥50	
Number of patients	2,288 (17.0)	6,036 (44.8)	5,162 (38.3)	
Age, years	64.4 (2.9)	64.6 (2.9)	64.7 (2.8)	0.55
Male	1,369 (59.8)	3,867 (64.1)	3,432 (66.5)	<0.001
Education				<0.001
College or university degree	252 (11.0)	529 (8.8)	425 (8.2)	
A/AS levels or equivalent or O levels/GCSE or CSE or equivalent	447 (20.9)	1,230 (20.4)	1,095 (21.2)	
NVQ or HND or HNC or equivalent or other professional qualifications	732 (32.0)	2,132 (35.3)	1,751 (33.9)	
None of the above	755 (33.0)	2,018 (33.4)	1,799 (34.9)	
Missing	72 (3.2)	127 (2.1)	92 (1.8)	
Townsend deprivation index	0.16 (3.50)	−0.57 (3.34)	−1.31 (3.05)	<0.001
Physical activity, MET-hours/week	26.5 (39.3)	33.4 (47.3)	41.6 (54.0)	<0.001
BMI, kg/m^2^	32.1 (5.9)	31.5 (5.4)	30.0 (4.8)	<0.001
*APOE ε4* carrier	526 (23.0)	1,493 (24.7)	1,241 (24.0)	0.37
Sun-exposure time in summer, hours/day	3.8 (2.4)	4.2 (2.5)	4.6 (2.4)	0.33
Ethnicity				<0.001
White	1,839 (80.4)	5,452 (90.3)	4,938 (95.7)	
Mixed	56 (2.5)	122 (2.0)	44 (0.9)	
Asian or Asian British	292 (12.8)	244 (4.0)	88 (1.7)	
Black or Black British	70 (3.1)	168 (2.8)	76 (1.5)	
Missing	31 (1.4)	50 (0.8)	16 (0.3)	
Blood collection season				<0.001
Dec–Feb	736 (32.2)	1,346 (22.3)	697 (13.5)	
Mar–May	893 (39.0)	1,885 (31.2)	1,064 (20.6)	
Jun–Aug	270 (11.8)	1,387 (23.0)	1,977 (38.3)	
Sep–Nov	389 (17.0)	1,418 (23.5)	1,424 (27.6)	
Smoking status				<0.001
Never smoker	899 (39.3)	2,437 (40.4)	2,048 (39.7)	
Past smoker	1,053 (46.0)	2,978 (49.3)	2,748 (53.2)	
Current smoker	308 (13.5)	557 (9.2)	318 (6.2)	
Missing	28 (1.2)	64 (1.1)	48 (0.9)	
Alcohol drinking				<0.001
Never or special occasions	984 (43.0)	2,070 (34.3)	1,419 (27.5)	
Monthly to weekly	958 (41.9)	2,990 (49.5)	2,828 (54.8)	
Daily	335 (14.6)	954 (15.8)	908 (17.6)	
Missing	11 (0.5)	22 (0.4)	7 (0.1)	
Sleep duration, hours/day				<0.001
≤6	667 (29.2)	1,495 (24.8)	1,141 (22.1)	
7–8	1,211 (52.9)	3,531 (58.5)	3,259 (63.1)	
≥9	367 (16.0)	928 (15.4)	720 (14.0)	
Missing	43 (1.9)	82 (1.4)	42 (0.8)	
Duration of type 2 diabetes, years	8.1 (8.7)	7.8 (8.6)	7.5 (8.2)	<0.001
Healthy diet score				<0.001
Quintile 1	738 (32.3)	1,638 (27.1)	1,207 (23.4)	
Quintile 2	494 (21.6)	1,251 (20.7)	1,058 (20.5)	
Quintile 3	421 (18.4)	1,273 (21.1)	1,111 (21.5)	
Quintile 4	289 (12.6)	961 (15.9)	884 (17.1)	
Quintile 5	289 (12.6)	813 (13.5)	842 (16.3)	
Missing	57 (2.5)	100 (1.7)	60 (1.2)	
Multivitamin intake	165 (7.2)	921 (15.3)	1,206 (23.4)	<0.001
Vitamin D supplement intake	39 (1.7)	184 (3.1)	241 (4.7)	<0.001
History of cardiovascular disease	610 (26.7)	1,348 (22.3)	1,012 (19.6)	<0.001
History of hypertension	1,971 (86.2)	5,125 (84.9)	4,230 (81.9)	<0.001
History of cancer	236 (10.3)	627 (10.4)	550 (10.7)	0.19
History of depression	312 (13.6)	761 (12.6)	619 (12.0)	0.14
Medications for diabetes				0.05
None	667 (29.2)	1,872 (31.0)	1,635 (31.7)	
Only oral drugs	1,222 (53.4)	3,232 (53.6)	2,755 (53.4)	
Insulin and others	399 (17.4)	932 (15.4)	772 (15.0)	
TC, mmol/L	4.5 (1.0)	4.4 (0.9)	4.3 (0.9)	<0.001
TGs, mmol/L	2.4 (1.4)	2.2 (1.2)	1.9 (1.0)	<0.001
LDL-C, mmol/L	2.7 (0.7)	2.6 (0.7)	2.5 (0.7)	<0.001
CRP, mg/L	3.9 (5.7)	3.4 (5.1)	2.8 (4.3)	<0.001
HbA1c, mmol/mol	53.0 (13.2)	51.7 (12.0)	50.3 (10.7)	<0.001

Data are presented as mean (SD) or *N* (%). Abbreviations: 25(OH)D, 25-hydroxyvitamin D; CRP, C-reactive protein; CSE, Certificate of Secondary Education; GCSE, General Certificate of Secondary Education; HbA1c, hemoglobin; HNC, Higher National Certificate; HND, Higher National Diploma; LDL-C, low-density lipoprotein cholesterol; NVQ, National Vocational Qualification; TC, total cholesterol; TG, triglyceride.

### Restricted cubic spline analysis

**Fig 1:**
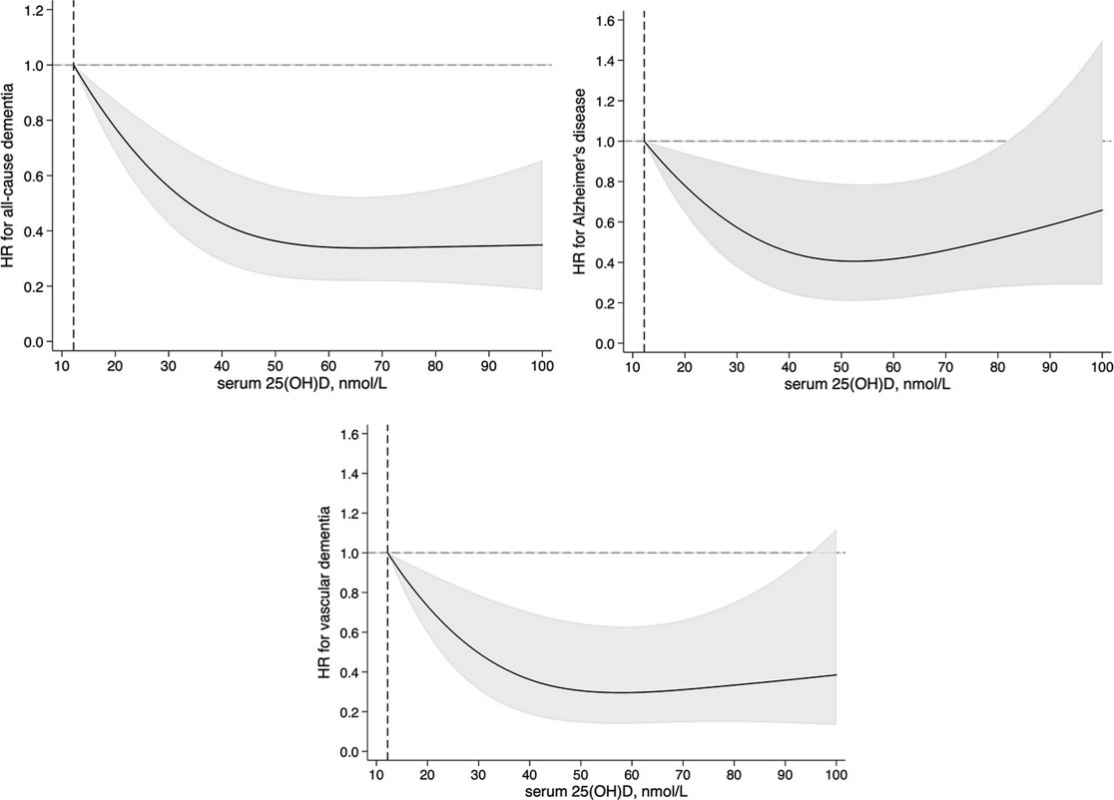
Multivariable adjusted hazard ratios (HRs) for risks of all-cause dementia, Alzheimer disease, and vascular dementia according to serum 25-hydroxyvitamin D (25[OH]D) level (nmol/L) among patients with type 2 diabetes (≥60 years old). HRs were adjusted for age at recruitment (years, continuous); sex (male, female); education (college or university degree, A/AS levels or equivalent or O levels/General Certificate of Secondary Education or Certificate of Secondary Education or equivalent, National Vocational Qualification or Higher National Diploma or Higher National Certificate or equivalent or other professional qualifications, none of the above); Townsend deprivation index (continuous); ethnicity (White, Mixed, Asian, Black); blood collection season (Dec–Feb, Mar–May, Jun–Aug, Sep–Nov); sun-exposure time in summer (hours/day, continuous); *APOE ε4* (carrier, non-carrier); BMI (kg/m^2^, continuous); alcohol intake (never or special occasions, monthly to weekly, daily); smoking status (never, past, current); physical activity (MET-hours/week, continuous); healthy diet score (≤3, >3); sleep duration (≤6, 7–8, ≥9 hours/day); multivitamin supplements (yes, no); diabetes duration (years, continuous); concentration of HbA1c (mmol/mol, continuous); medication for diabetes (none, only oral medicine, insulin and others); history of hypertension, cardiovascular disease, cancer, and depression (yes, no); medication for hypertension and cholesterol (yes, no); circulating total cholesterol (mmol/L, continuous); triglycerides (mmol/L, continuous); low-density lipoprotein cholesterol (mmol/L, continuous); and C-reactive protein (mg/L, continuous). Values beyond the 1th and 99th percentile of serum 25(OH)D were winsorized to minimize the potential impact of extreme values. *P*_nonlinearity_ < 0.001 for all-cause mortality, *P*_nonlinearity_ = 0.06 for Alzheimer disease, and *P*_nonlinearity_ = 0.007 for vascular dementia. Black curves are HRs, and grey zones are 95% CIs.

The restricted cubic spline analysis showed a nonlinear relationship between serum 25(OH)D and risk of all-cause dementia (*P*_nonlinearity_ < 0.001) and VD (*P*_nonlinearity_ = 0.007), and the nonlinear association reached borderline significance for AD (*P*_nonlinearity_ = 0.06), with a threshold at around a serum 25(OH)D value of 50 nmol/L for all the outcomes (Fig 1).

### Serum 25(OH)D and risk of all-cause dementia

With the adjustment for demographics, *APOE ε4* genotype, sun-exposure time, and blood collection season in Model 1, serum 25(OH)D concentrations were inversely associated with the risk of all-cause dementia in a dose-dependent manner (*P*_trend_ < 0.001). With the additional adjustment for diet and lifestyle factors in Model 2, and further adjustment for the pre-existing comorbidities, medication use, cardiometabolic biomarkers, and diabetes-related factors in Model 3, the associations were slightly attenuated. In Model 3, compared with the participants who were serum 25(OH)D severely deficient, the HR (95% CIs) for all-cause dementia was 0.59 (0.44–0.80) for those who were moderately deficient of serum 25(OH)D (25 to <50 nmol/L) and 0.41 (0.29–0.60) for those with serum 25(OH)D level ≥ 50 nmol/L (*P*_trend_ < 0.001) (Table 2). Further, the HR (95% CI) of all-cause dementia was 0.99 (0.98–0.99) per unit increment in serum 25(OH)D level in Model 3.

**Table 2:** Hazard ratios (95% confidence intervals) for all-cause dementia, Alzheimer disease, and vascular dementia according to serum 25-hydroxyvitamin D (25[OH]D) concentration among individuals with type 2 diabetes who were ≥60 years old.

Outcome and model	Serum 25(OH)D concentration (nmol/L)			*P* for trend	Per unit
	<25	25 to <50	≥50		
**All-cause dementia**					
Cases/person-years	78/18,878	128/51,214	77/44,338		283/114,430
Unadjusted	1.00	0.60 (0.45–0.79)	0.41 (0.30–0.57)	<0.001	0.98 (0.98–0.99)
Model 1	1.00	0.56 (0.42–0.75)	0.37 (0.26–0.53)	<0.001	0.98 (0.98–0.99)
Model 2	1.00	0.59 (0.44–0.79)	0.42 (0.29–0.60)	<0.001	0.99 (0.98–0.99)
Model 3	1.00	0.59 (0.44–0.80)	0.41 (0.29–0.60)	<0.001	0.99 (0.98–0.99)
**Alzheimer disease**					
Cases/person-years	26/18,999	43/51,431	32/44,487		101/114,917
Unadjusted	1.00	0.60 (0.37–0.98)	0.51 (0.31–0.86)	0.03	0.99 (0.98–1.00)
Model 1	1.00	0.57 (0.34–0.94)	0.49 (0.27–0.86)	0.03	0.99 (0.98–1.00)
Model 2	1.00	0.55 (0.33–0.92)	0.47 (0.26–0.86)	0.04	0.99 (0.98–1.00)
Model 3	1.00	0.58 (0.34–0.97)	0.50 (0.27–0.92)	0.06	0.99 (0.98–1.00)
**Vascular dementia**					
Cases/person-years	30/18,998	41/51,429	26/44,484		97/114,911
Unadjusted	1.00	0.50 (0.31–0.80)	0.36 (0.22–0.62)	0.001	0.98 (0.97–0.99)
Model 1	1.00	0.47 (0.29–0.77)	0.33 (0.18–0.58)	0.001	0.98 (0.97–0.99)
Model 2	1.00	0.50 (0.30–0.83)	0.37 (0.20–0.68)	0.004	0.98 (0.97–1.00)
Model 3	1.00	0.53 (0.32–0.88)	0.41 (0.22–0.77)	0.01	0.99 (0.97–1.00)

Model 1: Adjusted for age at recruitment (continuous, years), sex (male, female), education (college or university degree, A/AS levels or equivalent or O levels/General Certificate of Secondary Education or Certificate of Secondary Education or equivalent, National Vocational Qualification or Higher National Diploma or Higher National Certificate or equivalent or other professional qualifications, none of the above), Townsend deprivation index (continuous), ethnicity (White, Mixed, Asian, Black), blood collection season (Dec–Feb, Mar–May, Jun–Aug, Sep–Nov), sun-exposure time in summer (continuous, hours/day), and *APOE ε4* (carrier, non-carrier). Model 2: Model 1 + BMI (continuous, kg/m^2^), alcohol intake (never or special occasions, monthly to weekly, daily), smoking status (never, past, current), physical activity (continuous, MET-hours/week), healthy diet score (in quintiles), sleep duration (≤6, 7–8, ≥9 hours/day), and multivitamin supplements (yes, no). Model 3: Model 2 + diabetes duration (continuous, years); concentration of HbA1c (continuous, mmol/mol); medication for diabetes (none, only oral medicine, insulin and others); history of hypertension, cardiovascular disease, cancer, and depression (yes, no); medication for hypertension and cholesterol (yes, no); circulating total cholesterol (continuous, mmol/L); triglycerides (continuous, mmol/L); low-density lipoprotein cholesterol (continuous, mmol/L); and C-reactive protein (continuous, mg/L).

### Serum 25(OH)D and risk of AD and VD

Serum 25(OH)D concentration was inversely associated with risk of AD in a dose-dependent manner in Model 1 (*P*_trend_ = 0.03). The association was largely unchanged in Model 2 and Model 3. In the fully adjusted Model 3, compared with the participants who were serum 25(OH)D severely deficient, the HR (95% CI) for AD was 0.58 (0.34–0.97) for those who were moderately deficient and 0.50 (0.27–0.92) for those with serum 25(OH)D level ≥ 50 nmol/L (*P*_trend_ = 0.06) (Table 2).

In addition, compared with the participants who were serum 25(OH)D severely deficient, the HR (95% CI) for VD was 0.53 (0.32–0.88) for those who were moderately deficient and 0.41 (0.22–0.77) for those with serum 25(OH)D level ≥ 50 nmol/L (*P*_trend_ = 0.01) in Model 3 (Table 2).

### Subgroup analysis and sensitivity analyses

The results were largely consistent when analyses were stratified by sex, *APOE ε4* genotype, BMI, diabetes duration, and smoking status, although some of the associations did not reach statistical significance, probably due to reduced power (S2 Table). We did not find any significant interaction between serum 25(OH)D and the stratified factors on the risk of the outcomes of interest considering multiple testing.

The results were robust in sensitivity analyses that either excluded cases with less than 2 years of follow-up (S3 Table) or adjusted for eGFR, frailty factors (walking pace and hearing difficulty), serum calcium, or vitamin D supplements (S4 Table). When serum 25(OH)D levels were categorized into 4 groups, serum 25(OH)D ≥ 75 nmol/L was significantly associated with a lower risk of all-cause dementia compared with the reference group (<25 nmol/L), while the association did not reach statistical significance for AD or VD, possibly due to insufficient statistical power (only 8 AD and 8 VD cases were in the category of 25(OH)D ≥ 75 nmol/L) (S5 Table). In addition, we observed similar associations of serum 25(OH)D with risks of outcomes in the full cohort population aged ≥60 years. In the fully adjusted model, comparing 25(OH)D level ≥ 50 nmol/L with <25 nmol/L, the HRs (95% CIs) of all-cause dementia, AD, and VD were 0.52 (0.44–0.61), 0.59 (0.46–0.76), and 0.62 (0.45–0.86), respectively (S6 Table). Further, the observed associations were more pronounced among individuals with T2D (*P*_interaction_ = 0.002 for all-cause dementia, *P*_interaction_ = 0.08 for AD, *P*_interaction_ = 0.03 for VD; S7 Table).

## Discussion

### Summary of the findings

In this large cohort study, higher levels of serum 25(OH)D were significantly associated with a lower risk of all-cause dementia, AD, and VD among patients with T2D. The observed associations were independent of the traditional and potential risk factors of dementia, including diet and lifestyle factors, severity of diabetes, frailty, and *APOE ε4* genotype.

### Comparison with other studies

Although the relationship between circulating 25(OH)D concentrations and risk of dementia has been reported among general populations in numerous prospective studies, the results are inconsistent [4–7,25–27]. The Three-City Bordeaux cohort, with 177 all-cause dementia cases including 124 AD cases, showed that vitamin D deficiency was associated with a 2 times higher risk of developing all-cause dementia and AD among participants aged over 65 years [7]. Similarly, the Cardiovascular Health Study, including 171 dementia cases (102 AD cases), showed that a circulating serum 25(OH)D level less than 50 nmol/L was a risk factor for all-cause dementia and AD [6]. The Copenhagen City Heart Study, with 418 AD and 92 VD cases, showed that, in the general population, circulating 25(OH)D levels less than 25 nmol/L were associated with a higher risk of AD but not VD [4]. In contrast, the EPIDOS Toulouse Study found that vitamin D deficiency (<25 nmol/L) was a risk factor for non-AD dementia but not AD among older women [28]. Null associations between vitamin D status and cognitive function have also been reported [25–27]. The heterogeneity in these findings might be explained by limited statistical power, residual confounding, variations in 25(OH)D level measurement, and unstandardized definitions of dementia diagnosis across studies.

Among patients with diabetes, who have a high prevalence of vitamin D deficiency and heightened risk of developing dementia [13–16], the evidence regarding vitamin D status and cognitive function is scarce [17–19]. A cross-sectional study found lower levels of serum 25(OH)D among T2D patients with mild cognitive impairment assessed using the Montreal Cognitive Assessment score [17]. Another 2 case–control studies showed that serum 25(OH)D levels were associated with Mini-Mental State Examination and Montreal Cognitive Assessment score among patients with T2D [18,19]. However, no study has examined the association between circulation 25(OH)D levels and dementia among patients with diabetes. To the best of our knowledge, our study is the first investigation finding that higher levels of serum 25(OH)D are significantly associated with a lower risk of all-cause dementia, AD, and VD among individuals with T2D. In addition, we observed a nonlinear association between serum 25(OH)D and risk of all-cause dementia and VD, and the nonlinear association reached borderline significance for AD, with a threshold at around a serum 25(OH)D value of 50 nmol/L. While the causality of these associations cannot be determined from the study, our findings could provide the basis for an intervention protocol targeting vitamin D supplements in dementia prevention to maintain an efficient circulating vitamin D level.

### Potential mechanisms

Although the exact mechanism underlying the relationship between vitamin D and dementia risk for individuals with diabetes remains to be further elucidated, it is widely postulated to involve both neurodegenerative and vascular pathways. Experimental studies have shown that vitamin D could increase the clearance of amyloid plaques by stimulating macrophages [29,30], and vitamin D was also found to suppress macrophage migration among patients with diabetes [31]. Further, the pathology of AD involves amyloid-β-induced nitric oxide synthase, via disruption of the vitamin D receptor pathway [32]. In vivo, an increase in vitamin D receptor was observed in neurons of diabetic rats, which indicated that the vitamin D receptor signaling system could be a potential therapeutic target for diabetic neuropathy [33]. In addition, accumulating evidence has suggested that vitamin D may improve glycemic control, blood pressure, and lipid metabolism among patients with diabetes [34–36]; dysregulation of these processes is linked to cerebrovascular pathologies, such as stroke and white matter hyperintensities, that are well-known risk factors of vascular dementia [37–40]. Nevertheless, more mechanistic studies are warranted to further illuminate the potential mechanisms through which vitamin D plays a role in the prevention of dementia among patients with diabetes.

### Strengths and limitations

A major strength of our study is that the UK Biobank study has collected extensive phenotypic and genotypic data; hence, we could meticulously adjust for a wide range of potential confounding factors. Several potential limitations of our study deserve mention. First, although, a recent validation study has shown an 82.5% positive predictive value of the ascertainment of all-cause dementia using EHR data in the UK Biobank study [41], some dementia cases may not have been captured as participants with poor cognitive function have a higher risk of loss to follow-up [42]. In addition, the ICD code may not be sufficient to detect cases in an early stage or to classify the subtypes of dementia cases. Further, due to lack of information, we are unable to investigate the association between vitamin D level and the risk of mixed causes of dementia in the current study. Second, we used the baseline one-time measurement of serum 25(OH)D levels in the UK Biobank; hence, changes in 25(OH)D levels were not captured during follow-up, which may lead to non-differential misclassification bias. However, previous evidence has indicated that a single measurement of circulating vitamin D could provide a precise estimate of long-term circulating 25(OH)D concentration [43]. Third, the current study did not account for vitamin D binding proteins and vitamin D receptor genes [10,44]; hence, we are unable to assess the relationship between bioavailable vitamin D levels and dementia risk. Fourth, self-reported diet and lifestyle factor data were subject to measurement error, which may lead to misclassification bias. Fifth, as our study was limited to patients with T2D who were aged ≥60 years from the UK Biobank study, our results may not be directly generalizable to other populations. Finally, despite comprehensive adjustment for potential confounders, residual confounding cannot be completely ruled out due to the limitation of the observational study design.

### Public health implications and next steps for research

There has been wide implementation of specific guidelines for secondary prevention of complications in patients with diabetes, and death rates for 10 of 12 causes of death in patients with diabetes have declined, but death rates for dementia have increased [3]. This transition requires clinical and preventative strategies for dementia prevention among patients with diabetes. Patients with T2D are known to be at higher risk of being vitamin D deficient [13,14]. Our findings, if confirmed by replication, may have implications for dementia prevention strategies that target improving and maintaining serum vitamin D concentrations among patients with T2D. These findings also contribute towards the scientific basis for the development of intervention studies of dementia prevention among patients with T2D in the future.

## Conclusions

In a large cohort study of individuals with T2D who were ≥60 years, we found that higher concentrations of serum 25(OH)D were significantly associated with a lower risk of all-cause dementia, AD, and VD. The findings suggest the possibility that serum vitamin D screening among patients with T2D may be useful in dementia care and prevention.

## Supporting information

S1 Checklist STROBE Statement—checklist of items that should be included in reports of cohort studies. (DOCX)

S1 Fig Flowchart for the selection of the study population from the UK Biobank study. (TIF)

S1 Table Ascertainment of dementia cases in the UK Biobank study. (DOCX)

S2 Table Subgroup analyses of serum 25(OH)D concentrations and risk of dementia, Alzheimer disease, and vascular dementia according to sex, *apolipoprotein E (APOE) ε4*, body mass index (BMI), diabetes duration, sleep duration, and smoking status. Hazard ratios were adjusted for age at recruitment (continuous, years); sex (male, female); education (college or university degree, A/AS levels or equivalent or O levels/GCSE or Certificate of Secondary Education or equivalent, NVQ or HND or HNC or equivalent or other professional qualifications, none of the above); Townsend deprivation index (continuous); ethnicity (White, Mixed, Asian, Black); blood collection season (Dec–Feb, Mar–May, Jun–Aug, Sep–Nov); sun-exposure time in summer (continuous, hours/day); *APOE ε4* (carrier, non-carrier); BMI (continuous, kg/m^2^); alcohol intake (never or special occasions, monthly to weekly, daily); smoking status (never, past, current); physical activity (continuous, MET-hours/week); healthy diet score (in quintiles); sleep duration (≤6, 7–8, ≥9 hours/day); multivitamin supplements (yes, no); diabetes duration (continuous, years); concentration of HbA1c (continuous, mmol/mol); medication for diabetes (none, only oral medicine, insulin and others); history of hypertension, cardiovascular disease, cancer, and depression (yes, no); medication for hypertension and cholesterol (yes, no); circulating total cholesterol (continuous, mmol/L); triglycerides (continuous, mmol/L); low-density lipoprotein cholesterol (continuous, mmol/L); and C-reactive protein (continuous, mg/L). The stratum variable was not included in the model when stratifying by itself.
(DOCX)

S3 Table HRs (95% CIs) for all-cause dementia, Alzheimer disease, and vascular dementia according to serum 25(OH)D concentration among adults with type 2 diabetes aged ≥60 years after excluding the participants with less than 2 years of follow-up. Model 1: Adjusted for age at recruitment (continuous, years); sex (male, female), education (college or university degree, A/AS levels or equivalent or O levels/GCSE or Certificate of Secondary Education or equivalent, NVQ or HND or HNC or equivalent or other professional qualifications, none of the above), Townsend deprivation index (continuous), ethnicity (White, Mixed, Asian, Black), blood collection season (Dec–Feb, Mar–May, Jun–Aug, Sep–Nov), sun-exposure time in summer (continuous, hours/day), and *APOE ε4* (carrier, non-carrier). Model 2: Model 1 + BMI (continuous, kg/m^2^), alcohol intake (never or special occasions, monthly to weekly, daily), smoking status (never, past, current), physical activity (continuous, MET-hours/week), healthy diet score (in quintiles), sleep duration (≤6, 7–8, ≥9 hours/day), and multivitamin supplements (yes, no). Model 3: Model 2 + diabetes duration (continuous, years); concentration of HbA1c (continuous, mmol/mol); medication for diabetes (none, only oral medicine, insulin and others); history of hypertension, cardiovascular disease, cancer, and depression (yes, no); medication for hypertension and cholesterol (yes, no); circulating total cholesterol (continuous, mmol/L); triglycerides (continuous, mmol/L); low-density lipoprotein cholesterol (continuous, mmol/L); and C-reactive protein (continuous, mg/L).
(DOCX)

S4 Table HRs (95% CIs) for all-cause dementia, Alzheimer disease, and vascular dementia according to serum 25(OH)D concentration with further adjustment for frailty factors, kidney function, serum calcium level, and vitamin D supplements. Model 1: Hazard ratios were adjusted for age at recruitment (continuous, years); sex (male, female); education (college or university degree, A/AS levels or equivalent or O levels/GCSE or Certificate of Secondary Education or equivalent, NVQ or HND or HNC or equivalent or other professional qualifications, none of the above); Townsend deprivation index (continuous); ethnicity (White, Mixed, Asian, Black); blood collection season (Dec–Feb, Mar–May, Jun–Aug, Sep–Nov); sun-exposure time in summer (continuous, hours/day); *APOE ε4* (carrier, non-carrier); BMI (continuous, kg/m^2^); alcohol intake (never or special occasions, monthly to weekly, daily); smoking status (never, past, current); physical activity (continuous, MET-hours/week); healthy diet (in quintiles); sleep duration (≤6, 7–8, ≥9 hours/day); multivitamin supplements (yes, no); diabetes duration (continuous, years); concentration of HbA1c (continuous, mmol/mol); medication for diabetes (none, only oral medicine, insulin and others); history of hypertension, cardiovascular disease, cancer, and depression (yes, no); medication for hypertension and cholesterol (yes, no); circulating total cholesterol (continuous, mmol/L); triglycerides (continuous, mmol/L); low-density lipoprotein cholesterol (continuous, mmol/L); and C-reactive protein (continuous, mg/L). Model 2: Model 1 + eGRF (continuous, mL/min/1.73 m^2^). Model 3: Model 1 + usual walking pace (slow, steady average, brisk) and hearing difficulty (yes, no). Model 4: Model 1 + serum calcium (continuous, mmol/L). Model 5: Model 1 + vitamin D supplements (yes, no).
(DOCX)

S5 Table Hazard ratios (95% confidence intervals) for all-cause dementia, Alzheimer disease, and vascular dementia according to serum 25(OH)D concentration among patients with type 2 diabetes (≥60 years old). Model 1: Adjusted for age at recruitment (continuous, years), sex (male, female), education (college or university degree, A/AS levels or equivalent or O levels/GCSE or Certificate of Secondary Education or equivalent, NVQ or HND or HNC or equivalent or other professional qualifications, none of the above), Townsend deprivation index (continuous), ethnicity (White, Mixed, Asian, Black), blood collection season (Dec–Feb, Mar–May, Jun–Aug, Sep–Nov), sun-exposure time in summer (continuous, hours/day), and *APOE ε4* (carrier, non-carrier). Model 2: Model 1 + BMI (continuous, kg/m^2^), alcohol intake (never or special occasions, monthly to weekly, daily), smoking status (never, past, current), physical activity (continuous, MET-hours/week), healthy diet score (in quintiles), sleep duration (≤6, 7–8, ≥9 hours/day), and multivitamin supplements (yes, no). Model 3: Model 2 + diabetes duration (continuous, years); concentration of HbA1c (continuous, mmol/mol); medication for diabetes (none, only oral medicine, insulin and others); history of hypertension, cardiovascular disease, cancer, depression, and diabetes (yes, no); medication for hypertension and cholesterol (yes, no); circulating total cholesterol (continuous, mmol/L); triglycerides (continuous, mmol/L); low-density lipoprotein cholesterol (continuous, mmol/L); and C-reactive protein (continuous, mg/L).
(DOCX)

S6 Table Hazard ratios (95% confidence intervals) for all-cause dementia, Alzheimer disease, and vascular dementia according to serum 25(OH)D concentration among individuals ≥60 years old (*N* = 192,862). Model 1: Adjusted for age at recruitment (continuous, years), sex (male, female), education (college or university degree, A/AS levels or equivalent or O levels/GCSE or Certificate of Secondary Education or equivalent, NVQ or HND or HNC or equivalent or other professional qualifications, none of the above), Townsend deprivation index (continuous), ethnicity (White, Mixed, Asian, Black), blood collection season (Dec–Feb, Mar–May, Jun–Aug, Sep–Nov), sun-exposure time in summer (continuous, hours/day), and *APOE ε4* (carrier, non-carrier). Model 2: Model 1 + BMI (continuous, kg/m^2^), alcohol intake (never or special occasions, monthly to weekly, daily), smoking status (never, past, current), physical activity (continuous, MET-hours/week), healthy diet score (in quintiles), sleep duration (≤6, 7–8, ≥9 hours/day), and multivitamin supplements (yes, no). Model 3: Model 2 + diabetes duration (continuous, years); concentration of HbA1c (continuous, mmol/mol); medication for diabetes (none, only oral medicine, insulin and others); history of hypertension, cardiovascular disease, cancer, depression, and diabetes (yes, no); medication for hypertension and cholesterol (yes, no); circulating total cholesterol (continuous, mmol/L); triglycerides (continuous, mmol/L); low-density lipoprotein cholesterol (continuous, mmol/L); and C-reactive protein (continuous, mg/L).
(DOCX)

S7 Table Hazard ratios (95% confidence intervals) for all-cause dementia, Alzheimer disease, and vascular dementia according to serum 25(OH)D concentration stratified by pre-existing type 2 diabetes (T2D). Hazard ratios were adjusted for age at recruitment (continuous, years); sex (male, female); education (college or university degree, A/AS levels or equivalent or O levels/GCSE or Certificate of Secondary Education or equivalent, NVQ or HND or HNC or equivalent or other professional qualifications, none of the above); Townsend deprivation index (continuous); ethnicity (White, Mixed, Asian, Black); blood collection season (Dec–Feb, Mar–May, Jun–Aug, Sep–Nov); sun-exposure time in summer (continuous, hours/day); *APOE ε4* (carrier, non-carrier); BMI (continuous, kg/m^2^); alcohol intake (never or special occasions, monthly to weekly, daily); smoking status (never, past, current); physical activity (continuous, MET-hours/week); healthy diet score (in quintiles); sleep duration (≤6, 7–8, ≥9 hours/day); multivitamin supplements (yes, no); diabetes duration (continuous, years); concentration of HbA1c (continuous, mmol/mol); medication for diabetes (none, only oral medicine, insulin and others); history of hypertension, cardiovascular disease, cancer, and depression (yes, no); medication for hypertension and cholesterol (yes, no); circulating total cholesterol (continuous, mmol/L); triglycerides (continuous, mmol/L); low-density lipoprotein cholesterol (continuous, mmol/L); and C-reactive protein (continuous, mg/L).
(DOCX)

S1 Text Analysis plan. (DOCX)

## Abbreviations

25(OH)D: 25-hydroxyvitamin D
AD: Alzheimer disease
APOE: apolipoprotein E
BMI: body mass index
CI: confidence interval
CRP: C-reactive protein
CSE: Certificate of Secondary Education
eGFR: estimated glomerular filtration rate
EHR: electronic health record
GCSE: General Certificate of Secondary Education
HbA1c: hemoglobin A1c
HNC: Higher National Certificate
HND: Higher National Diploma
HR: hazard ratio
LDL-C: low-density lipoprotein cholesterol
NVQ: National Vocational Qualification
T2D: type 2 diabetes
TC: total cholesterol
TG: triglyceride
VD: vascular dementia
