# Supplementary material for: Association of serum 25-hydroxyvitamin D concentrations with risk of dementia among individuals with type 2 diabetes: A cohort study in the UK Biobank
Source: PLoS Med. 2022 Jan 13;19(1):e1003906. doi: 10.1371/journal.pmed.1003906 (PMC8797194; doi:10.1371/journal.pmed.1003906)
Supplement: S1 Table — (DOCX) [file pmed.1003906.s003.docx]

**S1 Table.** Ascertainment for dementia cases in the UK Biobank study

|  | **ICD-9** | **ICD-10** | **Self-reported in UK Biobank (field ID)** | **Medications (field ID and codes for medications)** |
| --- | --- | --- | --- | --- |
| ALL-cause dementia | 290.2, 290.3, 290.4, 291.2, 294.1,  331.0, 331.1, 331.2, 331.5 | A81.0, F00, F00.0, F00.1, F00.2, F00.9,  F01, F01.0, F01.1, F01.2, F01.3, F01.8, F01.9,  F02, F02.0, F02.1, F02.2, F02.3, F02.4, F02.8,  F03, F05.1, F10.6,  G30, G30.0, G30.1, G30.8, G30.9,  G31.0, G31.1, G31.8, I67.3 | 1263 | Prescriptions including memantine, donepezil, galantamine and rivastigmine; 20003 |
| Alzheimer’s disease | 331.0 | F00, F00.0, F00.1, F00.2, F00.9,  G30, G30.0, G30.1, G30.8, G30.9, |  |  |
| Vascular dementia | 290.4 | F01, F01.0, F01.1, F01.2, F01.3, F01.8, F01.9,  I67.3 |  |  |
