# Supplementary material for: Association of serum 25-hydroxyvitamin D concentrations with risk of dementia among individuals with type 2 diabetes: A cohort study in the UK Biobank
Source: PLoS Med. 2022 Jan 13;19(1):e1003906. doi: 10.1371/journal.pmed.1003906 (PMC8797194; doi:10.1371/journal.pmed.1003906)
Supplement: S2 Table — Hazard ratios were adjusted for age at recruitment (continuous, years); sex (male, female); education (college or university degree, A/AS levels or equivalent or O levels/GCSE or Certificate of Secondary Education or equivalent, NVQ or HND or HNC or equivalent or other professional qualifications, none of the above); Townsend deprivation index (continuous); ethnicity (White, Mixed, Asian, Black); blood collection season (Dec–Feb, Mar–May, Jun–Aug, Sep–Nov); sun-exposure time in summer (continuous, hours/day); APOE ε4 (carrier, non-carrier); BMI (continuous, kg/m2); alcohol intake (never or special occasions, monthly to weekly, daily); smoking status (never, past, current); physical activity (continuous, MET-hours/week); healthy diet score (in quintiles); sleep duration (≤6, 7–8, ≥9 hours/day); multivitamin supplements (yes, no); diabetes duration (continuous, years); concentration of HbA1c (continuous, mmol/mol); medication for diabetes (none, only oral medicine, insulin and others); history of hypertension, cardiovascular disease, cancer, and depression (yes, no); medication for hypertension and cholesterol (yes, no); circulating total cholesterol (continuous, mmol/L); triglycerides (continuous, mmol/L); low-density lipoprotein cholesterol (continuous, mmol/L); and C-reactive protein (continuous, mg/L). The stratum variable was not included in the model when stratifying by itself. (DOCX) [file pmed.1003906.s004.docx]

**S2 Table.** Subgroup analyses of the serum 25(OH)D concentrations with risk of dementia, Alzheimer’s disease, and vascular dementia according to sex, *Apolipoprotein E ε4* (*AOPE ε4*), body mass index (BMI), diabetes duration, sleep duration and smoking status^*^

|  | **Serum 25(OH)D concentrations (nmol/L)** | | | |
| --- | --- | --- | --- | --- |
|  | **<25** | **25-50** | **≥50** | ***P* for trend** |
| **All dementia** | | | | |
| Male |  |  |  |  |
| Cases | 52 | 90 | 54 |  |
| Model 1 | 1.00 | 0.59 (0.42-0.83) | 0.39 (0.26-0.57) | <0.001 |
| Model 2 | 1.00 | 0.58 (0.40-0.83) | 0.38 (0.24-0.60) | <0.001 |
| Female |  |  |  |  |
| Cases | 26 | 38 | 23 |  |
| Model 1 | 1.00 | 0.60 (0.36-0.98) | 0.45 (0.26-0.79) | 0.009 |
| Model 2 | 1.00 | 0.62 (0.36-1.05) | 0.47 (0.25-0.90) | 0.03 |
| *P*-interaction | 0.54 |  |  |  |
| *AOPEε4* carrier |  |  |  |  |
| Cases | 36 | 50 | 32 |  |
| Model 1 | 1.00 | 0.47 (0.31-0.72) | 0.36 (0.22-0.57) | <0.001 |
| Model 2 | 1.00 | 0.46 (0.29-0.74) | 0.30 (0.17-0.53) | <0.001 |
| *AOPEε4* non*-*carrier |  |  |  |  |
| Cases | 42 | 73 | 45 |  |
| Model 1 | 1.00 | 0.65 (0.44-0.95) | 0.45 (0.30-0.69) | <0.001 |
| Model 2 | 1.00 | 0.67 (0.45-1.01) | 0.51 (0.31-0.83) | 0.01 |
| *P*-interaction | 0.41 |  |  |  |
| BMI ≤30 kg/m^2^ |  |  |  |  |
| Cases | 30 | 56 | 38 |  |
| Model 1 | 1.00 | 0.61 (0.39-0.95) | 0.38 (0.23-0.61) | <0.001 |
| Model 2 | 1.00 | 0.64 (0.40-1.04) | 0.38 (0.22-0.67) | 0.001 |
| BMI >30 kg/m^2^ |  |  |  |  |
| Cases | 48 | 72 | 39 |  |
| Model 1 | 1.00 | 0.59 (0.41-0.86) | 0.46 (0.30-0.70) | <0.001 |
| Model 2 | 1.00 | 0.58 (0.39-0.86) | 0.47 (0.29-0.78) | 0.003 |
| *P*-interaction | 0.40 |  |  |  |
| Diabetes duration ≤7 years |  |  |  |  |
| Cases | 43 | 58 | 43 |  |
| Model 1 | 1.00 | 0.48 (0.33-0.72) | 0.41 (0.27-0.63) | <0.001 |
| Model 2 | 1.00 | 0.39 (0.26-0.60) | 0.28 (0.17-0.47) | <0.001 |
| Diabetes duration >7 years |  |  |  |  |
| Cases | 35 | 70 | 34 |  |
| Model 1 | 1.00 | 0.75 (0.50-1.13) | 0.42 (0.26-0.67) | <0.001 |
| Model 2 | 1.00 | 0.88 (0.57-1.36) | 0.61 (0.36-1.06) | 0.06 |
| *P*-interaction | 0.60 |  |  |  |
| Never smokers |  |  |  |  |
| Cases | 33 | 49 | 27 |  |
| Model 1 | 1.00 | 0.54 (0.35-0.84) | 0.35 (0.21-0.59) | <0.001 |
| Model 2 | 1.00 | 0.54 (0.33-0.87) | 0.36 (0.20-0.65) | 0.002 |
| Ever smokers |  |  |  |  |
| Cases | 43 | 78 | 48 |  |
| Model 1 | 1.00 | 0.66 (0.46-0.96) | 0.45 (0.30-0.69) | <0.001 |
| Model 2 | 1.00 | 0.67 (0.45-1.00) | 0.45 (0.28-0.72) | 0.001 |
| *P*-interaction | 0.49 |  |  |  |
| **Alzheimer’s disease**  **S;’** | | | | |
| Male |  |  |  |  |
| Cases | 13 | 26 | 24 |  |
| Model 1 | 1.00 | 0.68 (0.35-1.32) | 0.69 (0.35-1.35) | 0.43 |
| Model 2 | 1.00 | 0.63 (0.31-1.28) | 0.62 (0.28-1.39) | 0.41 |
| Female |  |  |  |  |
| Cases | 13 | 17 | 8 |  |
| Model 1 | 1.00 | 0.53 (0.26-1.10) | 0.31 (0.13-0.75) | 0.01 |
| Model 2 | 1.00 | 0.50 (0.23-1.10) | 0.32 (0.12-0.88) | 0.04 |
| *P*-interaction | 0.18 |  |  |  |
| *AOPEε4* carrier |  |  |  |  |
| Cases | 17 | 14 | 16 |  |
| Model 1 | 1.00 | 0.28 (0.14-0.57) | 0.38 (0.19-0.75) | 0.04 |
| Model 2 | 1.00 | 0.25 (0.12-0.55) | 0.29 (0.13-0.66) | 0.02 |
| *AOPEε4* non*-*carrier |  |  |  |  |
| Cases | 9 | 27 | 16 |  |
| Model 1 | 1.00 | 0.12 (0.53-0.24) | 0.75 (0.33-1.71) | 0.31 |
| Model 2 | 1.00 | 1.21 (0.54-2.70) | 0.88 (0.34-2.24) | 0.55 |
| *P*-interaction | 0.23 |  |  |  |
| BMI ≤30 kg/m^2^ |  |  |  |  |
| Cases | 11 | 23 | 19 |  |
| Model 1 | 1.00 | 0.68 (0.33-1.39) | 0.51 (0.24-1.07) | 0.08 |
| Model 2 | 1.00 | 0.80 (0.36-1.78) | 0.59 (0.24-1.45) | 0.23 |
| BMI >30 kg/m^2^ |  |  |  |  |
| Cases | 15 | 20 | 13 |  |
| Model 1 | 1.00 | 0.53 (0.27-1.03) | 0.49 (0.23-1.03) | 0.07 |
| Model 2 | 1.00 | 0.51 (0.25-1.07) | 0.55 (0.23-1.32) | 0.20 |
| *P*-interaction | 0.90 |  |  |  |
| Diabetes duration ≤7 years |  |  |  |  |
| Cases | 14 | 20 | 18 |  |
| Model 1 | 1.00 | 0.51 (0.26-1.01) | 0.53 (0.26-1.06) | 0.18 |
| Model 2 | 1.00 | 0.37 (0.18-0.78) | 0.35 (0.15-0.80) | 0.06 |
| Diabetes duration >7 years |  |  |  |  |
| Cases | 12 | 23 | 14 |  |
| Model 1 | 1.00 | 0.72 (0.36-1.45) | 0.50 (0.23-1.08) | 0.08 |
| Model 2 | 1.00 | 0.93 (0.43-2.01) | 0.74 (0.29-1.84) | 0.47 |
| *P*-interaction | 0.93 |  |  |  |
| Never smokers |  |  |  |  |
| Cases | 15 | 15 | 10 |  |
| Model 1 | 1.00 | 0.36 (0.18-0.74) | 0.29 (0.13-0.64) | 0.007 |
| Model 2 | 1.00 | 0.44 (0.20-0.96) | 0.43 (0.17-1.10) | 0.12 |
| Ever smokers |  |  |  |  |
| Cases | 9 | 28 | 21 |  |
| Model 1 | 1.00 | 1.14 (0.54-2.41) | 0.95 (0.44-2.09) | 0.72 |
| Model 2 | 1.00 | 1.10 (0.49-2.43) | 0.82 (0.34-2.00) | 0.46 |
| *P*-interaction | 0.05 |  |  |  |
| **Vascular dementia** | | | | |
| Male |  |  |  |  |
| Cases | 23 | 35 | 19 |  |
| Model 1 | 1.00 | 0.52 (0.31-0.88) | 0.31 (0.17-0.57) | <0.001 |
| Model 2 | 1.00 | 0.56 (0.31-0.99) | 0.37 (0.18-0.76) | 0.01 |
| Female |  |  |  |  |
| Cases | 7 | 6 | 7 |  |
| Model 1 | 1.00 | 0.35 (0.12-1.04) | 0.51 (0.18-1.45) | 0.39 |
| Model 2 | 1.00 | 0.37 (0.11-1.23) | 0.51 (0.14-1.82) | 0.50 |
| *P*-interaction | 0.53 |  |  |  |
| *AOPEε4* carrier |  |  |  |  |
| Cases | 14 | 17 | 8 |  |
| Model 1 | 1.00 | 0.42 (0.21-0.84) | 0.23 (0.10-0.55) | 0.002 |
| Model 2 | 1.00 | 0.43 (0.19-0.95) | 0.21 (0.07-0.57) | 0.004 |
| *AOPEε4* non*-*carrier |  |  |  |  |
| Cases | 16 | 23 | 18 |  |
| Model 1 | 1.00 | 0.54 (0.28-1.02) | 0.48 (0.24-0.94) | 0.07 |
| Model 2 | 1.00 | 0.64 (0.32-1.27) | 0.65 (0.29-1.47) | 0.43 |
| *P*-interaction | 0.18 |  |  |  |
| BMI ≤30 kg/m^2^ |  |  |  |  |
| Cases | 15 | 16 | 10 |  |
| Model 1 | 1.00 | 0.35 (0.17-0.70) | 0.20 (0.09-0.44) | <0.001 |
| Model 2 | 1.00 | 0.29 (0.13-0.65) | 0.15 (0.06-0.41) | <0.001 |
| BMI >30 kg/m^2^ |  |  |  |  |
| Cases | 15 | 25 | 16 |  |
| Model 1 | 1.00 | 0.66 (0.35-1.26) | 0.61 (0.30-1.23) | 0.19 |
| Model 2 | 1.00 | 0.77 (0.38-1.54) | 0.84 (0.36-1.93) | 0.70 |
| *P*-interaction | 0.04 |  |  |  |
| Diabetes duration ≤7 years |  |  |  |  |
| Cases | 15 | 19 | 10 |  |
| Model 1 | 1.00 | 0.46 (0.23-0.90) | 0.28 (0.12-0.61) | 0.003 |
| Model 2 | 1.00 | 0.37 (0.18-0.76) | 0.19 (0.07-0.49) | 0.001 |
| Diabetes duration >7 years |  |  |  |  |
| Cases | 15 | 22 | 16 |  |
| Model 1 | 1.00 | 0.55 (0.29-1.06) | 0.46 (0.23-0.93) | 0.06 |
| Model 2 | 1.00 | 0.67 (0.32-1.37) | 0.77 (0.33-1.79) | 0.71 |
| *P*-interaction | 0.89 |  |  |  |
| Never smokers |  |  |  |  |
| Cases | 11 | 11 | 9 |  |
| Model 1 | 1.00 | 0.36 (0.16-0.84) | 0.36 (0.15-0.86) | 0.06 |
| Model 2 | 1.00 | 0.32 (0.13-0.80) | 0.28 (0.10-0.83) | 0.06 |
| Ever smokers |  |  |  |  |
| Cases | 18 | 29 | 16 |  |
| Model 1 | 1.00 | 0.59 (0.33-1.06) | 0.37 (0.19-0.72) | 0.005 |
| Model 2 | 1.00 | 0.62 (0.33-1.17) | 0.41 (0.18-0.90) | 0.03 |
| *P*-interaction | 0.96 |  |  |  |

Model 1: unadjusted model.

Model 2: age at recruitment (continuous, years), sex (male, female), education (college or university degree, A/AS levels or equivalent or O levels/GCSEs, NVQ or HND or HNC or equivalent or other professional qualifications, none of the above), Townsend Deprivation Index (continuous), ethnicity (White, Mixed, Asian, Black), blood collection season (Dec-Feb, Mar-May, Jun-Aug, Sep-Nov), sun-exposure time in summer (continuous, hours/day), *AOPEε4* (carriers, non-carriers), BMI (continuous, kg/m^2^), alcohol intake (never or special occasions, monthly to weekly, daily), smoking status (never, past, current), physical activity (continuous, MET-hours/week), healthy diet score (in quintiles), sleep duration (≤6, 7-8, ≥9 hours/day), multi-vitamin supplements (yes, no), diabetes duration (continuous, years), concentrations of HbA1c (continuous, mmol/mol), medication for diabetes (none, only oral medicine, insulin and others), history of hypertension, cardiovascular disease, cancer and depression (yes, no), and medication for hypertension and cholesterol (yes, no), circulating total cholesterol (continuous, mmol/L), triglycerides (continuous, mmol/L), LDL-cholesterol (continuous, mmol/L) and C-reactive protein (continuous, mg/L). The strata variable was not included in the model when stratifying by itself.

^*^*P*-interaction were tested based on Model 2.
