# Supplementary material for: Association of serum 25-hydroxyvitamin D concentrations with risk of dementia among individuals with type 2 diabetes: A cohort study in the UK Biobank
Source: PLoS Med. 2022 Jan 13;19(1):e1003906. doi: 10.1371/journal.pmed.1003906 (PMC8797194; doi:10.1371/journal.pmed.1003906)
Supplement: S5 Table — Model 1: Adjusted for age at recruitment (continuous, years), sex (male, female), education (college or university degree, A/AS levels or equivalent or O levels/GCSE or Certificate of Secondary Education or equivalent, NVQ or HND or HNC or equivalent or other professional qualifications, none of the above), Townsend deprivation index (continuous), ethnicity (White, Mixed, Asian, Black), blood collection season (Dec–Feb, Mar–May, Jun–Aug, Sep–Nov), sun-exposure time in summer (continuous, hours/day), and APOE ε4 (carrier, non-carrier). Model 2: Model 1 + BMI (continuous, kg/m2), alcohol intake (never or special occasions, monthly to weekly, daily), smoking status (never, past, current), physical activity (continuous, MET-hours/week), healthy diet score (in quintiles), sleep duration (≤6, 7–8, ≥9 hours/day), and multivitamin supplements (yes, no). Model 3: Model 2 + diabetes duration (continuous, years); concentration of HbA1c (continuous, mmol/mol); medication for diabetes (none, only oral medicine, insulin and others); history of hypertension, cardiovascular disease, cancer, depression, and diabetes (yes, no); medication for hypertension and cholesterol (yes, no); circulating total cholesterol (continuous, mmol/L); triglycerides (continuous, mmol/L); low-density lipoprotein cholesterol (continuous, mmol/L); and C-reactive protein (continuous, mg/L). (DOCX) [file pmed.1003906.s007.docx]

**S5 Table.** Hazard ratios (95% confidence intervals) for all-cause dementia, Alzheimer’s disease, and vascular dementia according to serum 25(OH)D concentrations among patients with type 2 diabetes (≥60 years old)

|  | **Serum 25(OH)D concentrations (nmol/L)** | | | |  |
| --- | --- | --- | --- | --- | --- |
|  | **<25** | **25 to <50** | **50 to <75** | **≥75** | ***P* for trend** |
| **All-cause dementia** |  |  |  |  |  |
| Cases | 78 | 128 | 56 | 21 |  |
| Unadjusted | 1.00 | 0.60 (0.45-0.79) | 0.39 (0.28-0.56) | 0.47 (0.29-0.77) | <0.001 |
| Model 1 | 1.00 | 0.56 (0.42-0.75) | 0.36 (0.25-0.51) | 0.43 (0.26-0.71) | <0.001 |
| Model 2 | 1.00 | 0.60 (0.44-0.80) | 0.40 (0.27-0.59) | 0.51 (0.30-0.86) | <0.001 |
| Model 3 | 1.00 | 0.59 (0.44-0.80) | 0.40 (0.27-0.59) | 0.48 (0.28-0.82) | <0.001 |
| **Alzheimer’s disease** |  |  |  |  |  |
| Cases | 26 | 43 | 24 | 8 |  |
| Unadjusted | 1.00 | 0.60 (0.37-0.98) | 0.51 (0.29-0.88) | 0.54 (0.24-1.19) | 0.03 |
| Model 1 | 1.00 | 0.57 (0.35-0.95) | 0.48 (0.26-0.88) | 0.52 (0.22-1.21) | 0.04 |
| Model 2 | 1.00 | 0.58 (0.35-0.97) | 0.48 (0.26-0.90) | 0.53 (0.22-1.26) | 0.06 |
| Model 3 | 1.00 | 0.59 (0.35-1.00) | 0.51 (0.27-0.97) | 0.54 (0.22-1.31) | 0.08 |
| **Vascular dementia** |  |  |  |  |  |
| Cases | 30 | 41 | 18 | 8 |  |
| Unadjusted | 1.00 | 0.50 (0.31-0.80) | 0.33 (0.18-0.59) | 0.47 (0.22-1.03) | 0.001 |
| Model 1 | 1.00 | 0.47 (0.29-0.77) | 0.29 (0.16-0.55) | 0.41 (0.18-0.96) | 0.001 |
| Model 2 | 1.00 | 0.50 (0.30-0.82) | 0.34 (0.17-0.65) | 0.49 (0.20-1.16) | 0.009 |
| Model 3 | 1.00 | 0.53 (0.32-0.88) | 0.37 (0.19-0.73) | 0.53 (0.22-1.28) | 0.02 |

**Model 1:** age at recruitment (continuous, years), sex (male, female), education (college or university degree, A/AS levels or equivalent or O levels/GCSEs, NVQ or HND or HNC or equivalent or other professional qualifications, none of the above), Townsend Deprivation Index (continuous), ethnicity (White, Mixed, Asian, Black), blood collection season (Dec-Feb, Mar-May, Jun-Aug, Sep-Nov), sun-exposure time in summer (continuous, hours/day), and *AOPEε4* (carriers, non-carriers).

**Model 2:** Model 1 + BMI (continuous, kg/m^2^), alcohol intake (never or special occasions, monthly to weekly, daily), smoking status (never, past, current), physical activity (continuous, MET-hours/week), healthy diet score (in quintiles), sleep duration (≤6, 7-8, ≥9 hours/day), and multi-vitamin supplements (yes, no).

**Model 3:** Model 2 + diabetes duration (continuous, years), concentrations of HbA1c (continuous, mmol/mol), medication for diabetes (none, only oral medicine, insulin and others), history of hypertension, cardiovascular disease, cancer, depression or diabetes (yes, no), medication for hypertension or cholesterol (yes, no), circulating total cholesterol (continuous, mmol/L), triglycerides (continuous, mmol/L), LDL-cholesterol (continuous, mmol/L) and C-reactive protein (continuous, mg/L).
