# Supplementary material for: Association of serum 25-hydroxyvitamin D concentrations with risk of dementia among individuals with type 2 diabetes: A cohort study in the UK Biobank
Source: PLoS Med. 2022 Jan 13;19(1):e1003906. doi: 10.1371/journal.pmed.1003906 (PMC8797194; doi:10.1371/journal.pmed.1003906)
Supplement: S7 Table — Hazard ratios were adjusted for age at recruitment (continuous, years); sex (male, female); education (college or university degree, A/AS levels or equivalent or O levels/GCSE or Certificate of Secondary Education or equivalent, NVQ or HND or HNC or equivalent or other professional qualifications, none of the above); Townsend deprivation index (continuous); ethnicity (White, Mixed, Asian, Black); blood collection season (Dec–Feb, Mar–May, Jun–Aug, Sep–Nov); sun-exposure time in summer (continuous, hours/day); APOE ε4 (carrier, non-carrier); BMI (continuous, kg/m2); alcohol intake (never or special occasions, monthly to weekly, daily); smoking status (never, past, current); physical activity (continuous, MET-hours/week); healthy diet score (in quintiles); sleep duration (≤6, 7–8, ≥9 hours/day); multivitamin supplements (yes, no); diabetes duration (continuous, years); concentration of HbA1c (continuous, mmol/mol); medication for diabetes (none, only oral medicine, insulin and others); history of hypertension, cardiovascular disease, cancer, and depression (yes, no); medication for hypertension and cholesterol (yes, no); circulating total cholesterol (continuous, mmol/L); triglycerides (continuous, mmol/L); low-density lipoprotein cholesterol (continuous, mmol/L); and C-reactive protein (continuous, mg/L). (DOCX) [file pmed.1003906.s009.docx]

**S7 Table.** Hazard ratios (95% confidence intervals) for all-cause dementia, Alzheimer’s disease, and vascular dementia according to serum 25(OH)D concentrations stratified by pre-existing type 2 diabetes (T2D) ^*^

|  | **Serum 25(OH)D concentrations (nmol/L)** | | | |
| --- | --- | --- | --- | --- |
|  | **<25** | **25-50** | **≥50** | ***P* for trend** |
| **All dementia** | | | | |
| No T2D |  |  |  |  |
| Cases | 200 | 589 | 660 |  |
| Model 1 | 1.00 | 0.72 (0.62-0.85) | 0.64 (0.54-0.74) | <0.001 |
| Model 2 | 1.00 | 0.70 (0.60-0.83) | 0.55 (0.46-0.66) | <0.001 |
| With T2D |  |  |  |  |
| Cases | 78 | 128 | 77 |  |
| Model 1 | 1.00 | 0.60 (0.45-0.79) | 0.41 (0.30-0.57) | <0.001 |
| Model 2 | 1.00 | 0.59 (0.43-0.79) | 0.42 (0.29-0.60) | <0.001 |
| *P*-interaction | 0.002 |  |  |  |
| **Alzheimer’s disease**  **S;’** | | | | |
| No T2D |  |  |  |  |
| Cases | 70 | 238 | 283 |  |
| Model 1 | 1.00 | 0.84 (0.64-1.09) | 0.78 (0.60-1.01) | 0.08 |
| Model 2 | 1.00 | 0.79 (0.60-1.03) | 0.63 (0.47-0.85) | 0.001 |
| With T2D |  |  |  |  |
| Cases | 26 | 43 | 32 |  |
| Model 1 | 1.00 | 0.60 (0.37-0.98) | 0.51 (0.31-0.86) | 0.02 |
| Model 2 | 1.00 | 0.59 (0.35-0.99) | 0.51 (0.28-0.95) | 0.05 |
| *P*-interaction | 0.08 |  |  |  |
| **Vascular dementia** | | | | |
| No T2D |  |  |  |  |
| Cases | 35 | 122 | 131 |  |
| Model 1 | 1.00 | 0.86 (0.59-1.25) | 0.73 (0.50-1.05) | 0.06 |
| Model 2 | 1.00 | 0.94 (0.64-1.38) | 0.78 (0.51-1.17) | 0.14 |
| With T2D |  |  |  |  |
| Cases | 30 | 41 | 26 |  |
| Model 1 | 1.00 | 0.50 (0.31-0.80) | 0.36 (0.22-0.62) | <0.001 |
| Model 2 | 1.00 | 0.52 (0.31-0.86) | 0.40 (0.21-0.75) | 0.005 |
| *P*-interaction | 0.03 |  |  |  |

Model 1: unadjusted model.

Model 2: age at recruitment (continuous, years), sex (male, female), education (college or university degree, A/AS levels or equivalent or O levels/GCSEs, NVQ or HND or HNC or equivalent or other professional qualifications, none of the above), Townsend Deprivation Index (continuous), ethnicity (White, Mixed, Asian, Black), blood collection season (Dec-Feb, Mar-May, Jun-Aug, Sep-Nov), sun-exposure time in summer (continuous, hours/day), *AOPEε4* (carriers, non-carriers), BMI (continuous, kg/m^2^), alcohol intake (never or special occasions, monthly to weekly, daily), smoking status (never, past, current), physical activity (continuous, MET-hours/week), healthy diet score (in quintiles), sleep duration (≤6, 7-8, ≥9 hours/day), multi-vitamin supplements (yes, no), diabetes duration (continuous, years), concentrations of HbA1c (continuous, mmol/mol), medication for diabetes (none, only oral medicine, insulin and others), history of hypertension, cardiovascular disease, cancer and depression (yes, no), and medication for hypertension and cholesterol (yes, no), circulating total cholesterol (continuous, mmol/L), triglycerides (continuous, mmol/L), LDL-cholesterol (continuous, mmol/L) and C-reactive protein (continuous, mg/L).

^*^ *P*-interaction were based on Model 2.
