# Supplementary material for: Association of serum 25-hydroxyvitamin D concentrations with risk of dementia among individuals with type 2 diabetes: A cohort study in the UK Biobank
Source: PLoS Med. 2022 Jan 13;19(1):e1003906. doi: 10.1371/journal.pmed.1003906 (PMC8797194; doi:10.1371/journal.pmed.1003906)
Supplement: S1 Text — (DOCX) [file pmed.1003906.s010.docx]

**Analysis Plan**

**Date:**

28^th^ Nov 2020

**Submitter:**

Tingting Geng

**Last Author:**

Gang Liu

**Proposal Title:**

Association of Serum 25-Hydroxyvitamin D Concentrations with Risk of Dementia among Individuals with Type 2 Diabetes

**Background:**

Increasing evidence has indicated a decline rates of vascular disease mortality, which leads to a diversification of diabetes related mortality. The UK National Statistics mortality data showed that ten causes of death among diabetes patients declined; however, death rates due to dementia has increased. Therefore, the strategies for dementia prevention should be particularly emphasized among individuals with diabetes. Although some epidemiological studies have linked lower vitamin D concentrations with risk of dementia, the evidence regarding the relationship between vitamin D status and dementia risk among patients with T2D is limited. Moreover, compared with the general populations, individuals with diabetes are particularly susceptible to both vitamin D deficiency, and increased risk of developing dementia.

**Hypothesis:**

We hypothesize that circulating vitamin D concentrations is associated with risk of dementia among individuals with type 2 diabetes.

**Study design:** Prospective cohort study

**Exposures:** Circulating vitamin D levels

**Outcomes:**

Incidence of all-cause dementia, Alzheimer’s disease, and vascular dementia

**Covariates:**

Age at recruitment (years), sex (men, women), education (college or university degree, A/AS levels or equivalent or O levels/GCSEs or equivalent or other professional qualifications, none of the above), socio-economic status (Townsend Deprivation Index, continuous), ethnicity (White, Asian, Black, others), blood collection season (Dec-Feb, Mar-May, Jun-Aug, Sep-Nov), sun-exposure time in summer (continuous, hours/day), AOPEε4 genotype (carriers, non-carriers), BMI (kg/m2), alcohol intake (never, special occasions, 1-3 times/month, 1-2 times/week, 3-4 times/week, daily), smoking status (never, past, current), physical activity (MET-hours/week), healthy diet score (continuous), sleep duration (≤6, 7-8, ≥9 hours/day), multi-vitamin supplements (yes, no), diabetes duration (continuous, years), concentrations of glycated hemoglobin A1c (HbA1c; continuous, mmol/L), medication for diabetes (none, only oral medicine, insulin, others), history of hypertension, cardiovascular disease, cancer, or depression (yes, no), medication for hypertension or cholesterol (yes, no), clinical biomarkers, and frailty markers.

**Statistical analysis:**

1. We will examine the associations of circulating vitamin D levels with risk of dementia among patients with type 2 diabetes.

2. We will examine the interactions between circulating vitamin D levels with stratified factors on the risk of dementia.

3. Cox proportional hazards regression models will be used to estimate hazard ratios and 95% Cis.

4. Restricted cubic spline analysis will be used to assess the shape between vitamin D levels and dementia risk.
